# Locoregional recurrences after transanal total mesorectal excision of rectal cancer during implementation

**DOI:** 10.1002/bjs.11525

**Published:** 2020-04-04

**Authors:** S. E. van Oostendorp, H. J. Belgers, B. T. Bootsma, J. C. Hol, E. J. T. H. Belt, W. Bleeker, F. C. Den Boer, A. Demirkiran, M. S. Dunker, H. F. J. Fabry, E. J. R. Graaf, J. J. Knol, S. J. Oosterling, G. D. Slooter, D. J. A. Sonneveld, A. K. Talsma, H. L. Van Westreenen, M. Kusters, R. Hompes, H. J. Bonjer, C. Sietses, J. B. Tuynman

**Affiliations:** ^1^ Department of Surgery Amsterdam UMC, Location VUmc Amsterdam the Netherlands; ^2^ Department of Surgery Amsterdam UMC, Location AMC, Cancer Centre Amsterdam Amsterdam the Netherlands; ^3^ Zuyderland Medical Centre, Sittard‐Geleen and Heerlen Dordrecht the Netherlands; ^4^ Albert Schweitzer Hospital Dordrecht the Netherlands; ^5^ Wilhelmina Hospital Assen the Netherlands; ^6^ Zaans Medical Centre Zaandam the Netherlands; ^7^ Rode Kruis Hospital Beverwijk the Netherlands; ^8^ Noord West Hospital Alkmaar the Netherlands; ^9^ Bravis Hospital Roosendaal the Netherlands; ^10^ IJsselland Hospital Cappelle aan den Ijssel the Netherlands; ^11^ Maxima Medical Centre Veldhoven the Netherlands; ^12^ Deventer Hospital Deventer the Netherlands; ^13^ Isala Clinic Zwolle the Netherlands; ^14^ Dijklander Hospital Hoorn the Netherlands; ^15^ Gelderse Vallei Hospital Ede the Netherlands; ^16^ Spaarne Hospital Hoofddorp the Netherlands; ^17^ Department of Abdominal Surgery, Jessa Hospital Hasselt Belgium

## Abstract

**Background:**

Transanal total mesorectal excision (TaTME) has been proposed as an approach in patients with mid and low rectal cancer. The TaTME procedure has been introduced in the Netherlands in a structured training pathway, including proctoring. This study evaluated the local recurrence rate during the implementation phase of TaTME.

**Methods:**

Oncological outcomes of the first ten TaTME procedures in each of 12 participating centres were collected as part of an external audit of procedure implementation. Data collected from a cohort of patients treated over a prolonged period in four centres were also collected to analyse learning curve effects. The primary outcome was the presence of locoregional recurrence.

**Results:**

The implementation cohort of 120 patients had a median follow up of 21·9 months. Short‐term outcomes included a positive circumferential resection margin rate of 5·0 per cent and anastomotic leakage rate of 17 per cent. The overall local recurrence rate in the implementation cohort was 10·0 per cent (12 of 120), with a mean(s.d.) interval to recurrence of 15·2(7·0) months. Multifocal local recurrence was present in eight of 12 patients. In the prolonged cohort (266 patients), the overall recurrence rate was 5·6 per cent (4·0 per cent after excluding the first 10 procedures at each centre).

**Conclusion:**

TaTME was associated with a multifocal local recurrence rate that may be related to suboptimal execution rather than the technique itself. Prolonged proctoring, optimization of the technique to avoid spillage, and quality control is recommended.

## Graphical abstract

During the learning curve, the transanal total mesorectal excision procedure is associated with a high multifocal local recurrence rate, which appears to be related to suboptimal execution rather than the technique, and necessitates prolonged proctoring, optimization of the technique avoiding spillage, participation in controlled clinical trials with surgical quality control, and centralization until oncological safety is demonstrated.

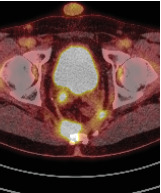

Defines a learning curve

## Abstract (es)

**Antecedentes:**

La escisión total del mesorrecto por vía transanal (*Transanal Total Mesorectal Excision* **,** TaTME) se ha propuesto como abordaje quirúrgico en pacientes con cáncer de recto medio e inferior. La técnica TaTME se ha introducido en los Países Bajos mediante un proceso de formación estructurado que incluye la supervisión. Este estudio evaluó el porcentaje de recidiva local durante la fase de implementación de TaTME.

**Métodos:**

Se recogieron los resultados oncológicos de los primeros 10 procedimientos realizados mediante TaTME en cada uno de los 12 centros participantes como parte de una auditoría externa de implementación del procedimiento. Se reunió una cohorte más amplia de pacientes procedentes de 4 centros para analizar los efectos de la curva de aprendizaje. El criterio de valoración principal fue la presencia de recidiva locorregional.

**Resultados:**

La cohorte de implementación de 120 pacientes tuvo una mediana de seguimiento de 21,9 meses. Los resultados a corto plazo incluyeron una tasa del margen de resección circunferencial positivo del 5% y una tasa de fuga anastomótica del 17,4%. La tasa global de recidiva local en la cohorte de implementación fue del 10% (12/120) con un intervalo medio de recidiva de 15,2 (DE 7) meses. El patrón de recidiva local fue multifocal en 8 de 12 casos (67%). En la cohorte ampliada (*n* = 266), la tasa global de recidiva fue del 5,6% (4,0%, excluyendo a los primeros 10 pacientes).

**Conclusión:**

TaTME se asoció con un porcentaje de recidiva local multifocal que puede relacionarse con una ejecución subóptima, más que con la técnica en sí. Se recomienda una supervisión prolongada, la optimización de la técnica para evitar la diseminación tumoral, así como un control de calidad.

## Introduction

The transanal total mesorectal excision (TaTME) technique has been introduced for patients with low rectal cancer, with the aim of improving clinical outcomes, such as a greater degree of radical resection, lower rates of anastomotic leakage, more sphincter‐saving procedures, better functional results and, most importantly, similar or lower local recurrence rates^1^, ^2^. Direct visualization facilitates purse‐string suture placement. The technique has been met with tremendous enthusiasm in the colorectal surgical community, and more than 300 centres worldwide have implemented the technique^3^. In expert centres, TaTME is associated with promising pathological and clinical outcomes^4^, ^5^, ^6^, ^7^, ^8^. The first long‐term outcome data from two expert centres showed a favourable low recurrence rate of 2 per cent after 3 years^9^.

Despite these positive results, it is also acknowledged that TaTME is a difficult technique and has a long learning curve with associated morbidity^10^, ^11^. The international TaTME registry^3^ and a systematic review^4^ have shown that widespread adoption results in less favourable clinical outcomes than reported in the initial cohorts treated in expert centres. The TaTME registry^3^, representing more than 300 centres voluntarily entering data, recorded an anastomotic failure rate of 15·6 per cent among 1594 patients, which is higher than rates from expert centres. In addition, a population‐based study^12^ documented an overall morbidity rate of 42·3 per cent, anastomotic leakage in 16·0 per cent and a circumferential resection margin (CRM)‐positive rate of 4·4 per cent. These latter studies show that the promise of TaTME has not yet been met on a large scale.

The long‐term oncological safety of TaTME remains to be proven. Although the first report with long‐term outcome data showed a low level of local recurrence, the question remains whether such results can be achieved with more widespread adoption of TaTME^9^. As TaTME is substantially different from abdominal techniques in terms of open access to the tumour, purse‐string closure and a subsequent endoluminal approach to the mesorectal dissection, it is especially important to assess long‐term outcomes properly. RCTs such as COLOR III^13^ and GRECCAR 11^14^ are investigating long‐term outcomes of TaTME, and are currently including patients. Recently, concern has been raised by the first report^15^ of national Norwegian data which showed an increase in the incidence of local recurrence with an extensive or multifocal pattern following TaTME, leading to a national halt to TaTME^16^.

In the Netherlands, a structured training pathway, including proctoring sessions by dedicated trainers, has been set up to ensure safe implementation of TaTME and minimization of learning curve effects^17^. A collective review of the short‐term outcomes of the first ten patients in 12 proctored centres revealed a major morbidity rate of 19·2 per cent and involved CRMs in 5·0 per cent of patients^17^. The aim of the present study was to evaluate the oncological outcomes of the initial patients who underwent TaTME within the structured training pathway. In addition, a cohort treated over a prolonged period after the implementation of TaTME in four high‐volume centres was evaluated to analyse learning curve effects in terms of local recurrence rates.

## Methods

### Structured training pathway

The structured training pathway was set up in the Netherlands in 2014 as a programme for postgraduate colorectal surgeons in centres with an annual volume of total mesorectal excision (TME) surgery for rectal cancer of 20 procedures or more and with known proficiency in laparoscopic TME. The clinical data from patients in the structured training pathway were collected prospectively, as described previously^17^. The first five procedures were discussed with and assisted by an experienced proctor, after which the following procedures were performed independently. The first ten patients in each of the centres that completed the structured training pathway were included to evaluate clinical outcomes during the implementation of TaTME^17^. In addition, a larger cohort of patients from four centres that continued TaTME after training, with a procedure volume greater than 45, was collected to assess learning curve effects. Long‐term clinical data were obtained as part of an external audit to assure high quality and completeness of the data set. The anonymized operative notes and full imaging reports of locoregional recurrences were obtained and audited by senior TaTME surgeons. All patients consented to a TaTME procedure as required under the Dutch national patient–physician relation regulations. The Medical Ethics Review Board of Amsterdam UMC, Location VUmc, approved the study and waived the need for additional informed consent for the present study.

### Outcomes

The primary outcome of this study was the incidence of local recurrence confirmed by either imaging (MRI, CT or PET–CT) and/or pathology (biopsy, salvage surgery). A local recurrence was defined as a mass in the pelvis with a biopsy positive for adenocarcinoma, or growth on sequential imaging in the absence of histopathological confirmation. A multifocal local recurrence was defined by the presence of two or more separate foci of recurrence in the pelvic area, as seen on MRI or PET–CT. Secondary outcomes included location of local recurrence and distant metastasis, treatment of recurrence and distant metastasis, and overall mortality. All potential risk factors were evaluated for an association with recurrence. Pelvic sepsis was defined by the occurrence of early anastomotic leakage, early pelvic abscess or late complications (leakage, abscess or presacral sinus occurring more than 30 days after operation)^18^. Complications were graded according to the Clavien–Dindo classification^19^. Rectal perforation, purse‐string failure and an insufficient anastomosis requiring reinforcement or refashioning were deemed to increase the risk of spillage of tumour cells into the pelvis. A positive CRM was defined by the presence of tumour cells 1 mm or less from the circumferential plane.

### Statistical analysis

Categorical data are shown as number with percentage, whereas continuous outcomes are recorded as mean(s.d.) or median (range). Dichotomous and categorical values were analysed using Pearson's χ^2^ test or Fisher's exact test. Comparison of continuous data was done using the independent Student's *t* test, or Mann–Whitney *U* test if the data were not distributed normally.

Univariable logistic regression analysis was performed to identify potential risk factors for local recurrence. Multivariable analysis was not possible because the event rate did not exceed the threshold for entry of multiple univariable significant predictors into a multivariable model. Case–control analysis between the present TaTME group and the laparoscopic TME group from the original COLOR II study was performed by matching sex, age, tumour height, neoadjuvant chemoradiotherapy, type of procedure (low anterior resection or abdominoperineal resection) and pathological risk factors, R1 and CRM and pT4 category^20^, ^21^. Patients with a final pT4 category or positive margins were excluded to enable evaluation of the technique as a potential individual risk factor for recurrence. For all tests, two‐sided *P ≤* 0·050 was considered statistically significant. Statistical analyses were done using SPSS® version 24 for Windows® and Mac® (IBM, Armonk, New York, USA).

## Results

### Baseline characteristics and clinical outcomes

A cohort of 120 patients, comprising the first ten patients in each of 12 centres who underwent TaTME between March 2015 and October 2018, was included. Median follow‐up was 21·9 (range 2·0–46·7) months. The median interval between the first and tenth procedures in each hospital was 12·5 (range 3·5–35·5) months. Baseline characteristics have been published previously and are shown in *Table* 1, 17.

**Figure 1:**
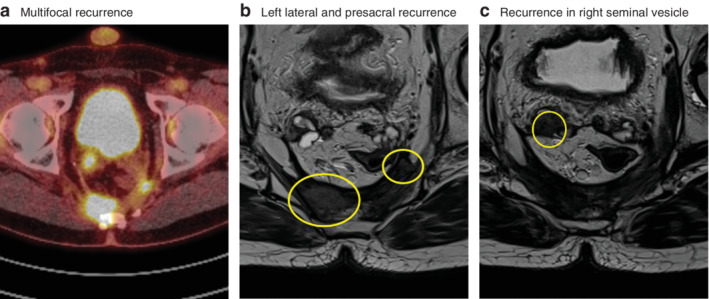
Images from a patient with multifocal recurrence after transanal total mesorectal excision **a** PET images showing multifocal recurrence. **b**,**c** T2‐weighted axial MRI images showing left lateral and presacral local recurrence (**b**) and recurrence in right seminal vesicle (**c**).

**Table 1:** Patient characteristics

	No. of patients*(*n* = 120)
**Age (years)** †	65·4(9·6)
**Sex ratio (M** : **F)**	91 : 29
**BMI (kg/m** ^**2**^ **)** †	26·9(4·1)
**ASA fitness grade**	
I	26 (21·7)
II	77 (64·2)
III	17 (14·2)
**Tumour height from anal verge (cm)** †	6·9(3·1)
**Clinical tumour category**	
(y)cT1	7 (5·8)
(y)cT2	24 (20·0)
(y)cT3	89 (74·2)
**Clinical node category**	
cN0	52 (43·3)
cN1	44 (36·7)
cN2	24 (20·0)
**Persistent MRF+ after RT** ‡	6 (5·0)
**Preoperative therapy**	
None	43 (35·8)
RT	41 (34·2)
CRT	36 (30·0)
**Transanal total mesorectal excision**	
Low anterior resection	110 (91·7)
Intersphincteric resection	10 (8·3)

* With percentages in parentheses unless indicated otherwise;

† values are mean(s.d.).

‡ All patients with a persistent theatened mesorectal fascia (MRF+) initially had cT3 tumours (3 anterior, 2 lateral, 1 unknown). RT, radiotherapy; CRT, chemoradiotherapy.

Short‐term outcomes are summarized in *Table* 2. The overall 30‐day morbidity rate was 45·0 per cent, including an anastomotic leakage rate of 17 per cent and pelvic sepsis in 17·5 per cent. The involved CRM rate was 5·0 per cent; no patient had an involved distal resection margin. The quality of the specimen was rated as complete in 89·2 per cent of procedures and nearly complete in 10·8 per cent; none of the specimens were considered incomplete.

**Table 2:** Short‐term clinicopathological outcomes

	No. of patients(*n* = 120)
**Intraoperative events**	
Purse‐string failure	1 (0·8)
Perforation	1 (0·8)
Reinforcement	3 (2·5)
**30‐day mortality**	0 (0)
**30‐day overall morbidity**	54 (45·0)
**Major morbidity (Clavien–Dindo grade ≥ III)**	23 (19·2)
**30‐day anastomotic leakage**	17 of 98 (17)
**Pelvic sepsis (early leak, abscess and late sinus)** *	21 (17·5)
**Pathological tumour category**	
(y)pT0	11 (9·2)
(y)pT1	16 (13·3)
(y)pT2	34 (28·3)
(y)pT3	59 (49·2)
(y)pT4	0 (0)
**Quality of specimen (Quirke)** *	
Complete	107 (89·2)
Nearly complete	13 (10·8)
Incomplete	0 (0)
**CRM involvement ≤ 1 mm**	6 (5·0)
**DRM involvement < 5 mm**	0 (0)

Values in parentheses are percentages.

* All patients (anastomosis and colostomy). CRM, circumferential resection margin; DRM, distal resection margin.

### Long‐term outcomes

Long‐term outcomes are shown in *Table* 3. Twelve of 120 patients (10·0 per cent) developed local recurrence, which was multifocal in eight patients. The median interval to local recurrence was 15·9 months, ranging from 6·0 to 26·4 months (*Table* 4). The recurrences were located presacrally (2), anterior (1), at the rectal stump (1) or in multiple regions in the pelvis (8) (*Fig*. 1). Nine of the 12 patients with local recurrence presented with or developed distant metastasis, whereas only 14 of 108 patients without local recurrence had distant metastases diagnosed (*P* < 0·001).

**Table 3:** Long‐term outcomes

	No. of patients*(*n* = 120)
**Follow‐up (months)**	
Mean(s.d.)	23·4(9·5)
Median (range)	21·9 (2·0–46·7)
**Local recurrence (total)**	12 (10·0)
**Multifocal local recurrence**	8 of 12 (67)
**Interval to local recurrence (months)** †	15·2(7·0)
**Overall distribution of disease (recurrence and metastasis)**	
Isolated local	3 (12)
Local + liver	4 (15)
Local + lung	2 (8)
Local + liver + lung	2 (8)
Local + lung + peritoneal + brain	1 (4)
Liver + lung	4 (15)
Isolated liver	5 (19)
Isolated lung	5 (19)
**Disease‐free surival**	94 (78·3)
**Overall survival**	115 (95·8)

* With percentages in parentheses unless indicated otherwise;

† values are mean(s.d.).

**Table 4:** Location and treatment of local recurrences

	No. of patients*(*n* = 12)
**Interval to local recurrence (months)**	
Mean(s.d.)	15·2(7·0)
Median (range)	15·9 (6·0–26·4)
**Location**	
Presacral	2
Anterior	1
Rectal stump	1
Multiple sites	8
**Focality (no. of sites)**	
1	4
2	4
3	4
**Treatment**	
Exenteration†	4
CRS + HIPEC	1
Abdominoperineal resection + IORT	1
Palliative chemotherapy	5
Further CRT; multivisceral resection planned	1

* Unless indicated otherwise.

† Also intraoperative radiotherapy (IORT) in one patient. CRS + HIPEC, cytoreductive surgery and hyperthermic intraperitoneal chemotherapy; CRT, chemoradiotherapy.

The local recurrences were distributed over the 12 participating sites as follows: three in one centre, two in three centres, one in three centres and none in five centres. There was no relationship between the time to include ten procedures and the incidence of local recurrence.

Details of the 12 patients who developed local recurrence are shown in *Table* 5. Two patients initially presented with a synchronous liver metastasis which was treated by a liver‐first approach. One of these developed lung metastasis simultaneous with the local recurrence. Pathological examination showed two poorly differentiated tumours, and three patients had an involved margin, one due to perineural growth that intersected the circumferential plane.

**Table 5:** Details of patients with local recurrence

Patient no.	Baseline data (sex, age, tumour height, cTNM stage)	Neoadjuvant treatment, MRF status*	Surgery	Anastomotic leakage	Pathological stage	Differentiation	CRM (mm)	Follow‐up details	Treatment	Outcome
1	M, 68 years, 2 cm from AV, cT2 N0 M0	No, MRF–	LAR + diversion	No	pT3 N0	W/M	10	18 months LR (multifocal) + M (hepatic)	Metastasectomy, APR + IORT	M+ (pulmonary), palliative chemotherapy. Alive at 35 months
2	F, 50 years, 8 cm from AV, cT3 N2 M1 (hepatic)	CRT, MRF–	Liver‐first laparoscopic segmentectomy VI + VII. LAR + diversion	Yes	pT3 N0	W/M	5	19 months LR (unifocal)	Exenteration	Further recurrence after 3 months. Alive at 28 months
3	F, 54 years, 3 cm from AV, cT3 N0 M0	No, MRF–	LAR + diversion	No	pT3 N0	W/M	3	26 months LR (unifocal) + M (hepatic)	Metastasectomy, exenteration	Disease‐free. Alive at 39 months
4	M, 65 years, 4 cm from AV, cT3 N1 M0	CRT, MRF–	LAR, no stoma	No	pT2 N1	Poor	3	12 months LR (multifocal)	Exenteration (R1)	M+ (hepatectomy) after 5 months, palliative chemotherapy. Died 36 months after TME
5	M, 55 years, 8 cm from AV, cT3 N0 M0	5 × 5, MRF–	LAR, no stoma	Yes	pT3 N1	W/M	4	7 months LR (multifocal) + M (hepatic)	Palliative	Alive at 23 months
6	M, 40 years, 8·3 cm from AV, cT3 N2 M0	CRT, MRF–	LAR + diversion	No	pT3 N2	W/M	7	14 months LR (multifocal) + M (pulmonary)	Further CRT, systemic chemotherapy	Progression, palliative. Alive at 25 months
7	M, 85 years, 2 cm from AV, cT3 N2 M0	CRT, MRF–	LAR + colostomy	No	pT3 N0	Poor	0	10 months LR (unifocal)	Palliative	Died 15 months after TME
8	M, 51 years, 5 cm from AV, cT3 N2 M1 (hepatic)	CRT, MRF–	Liver‐first laparoscopic segmentectomy IVb. LAR + diversion	No	pT3 N1	W/M	< 1	8 months LR (multifocal) + M (pulmonary)	Pulmonary RT. Response to induction chemotherapy. Recurrent M+ (pulmonary)	Palliative chemotherapy. Alive at 34 months
9	F, 54 years, 3 cm from AV, cT3 N1 M0	CRT, MRF–	LAR + diversion	No	pT3 N1	W/M	> 10	25 months LR (multifocal)	Induction chemotherapy + further CRT. CRS + HIPEC (R0)	Alive 36 at months
10	M, 60 years, 7 cm from AV, cT3 N1 M0	5 × 5, MRF–	LAR + diversion; air leak reinforced by sutures	Yes	pT3 N0	W/M	> 10	20 months LR (multifocal)	Induction chemotherapy + further CRT. Exenteration (R0)	Alive at 22 months
11	F, 75 years, 5 cm from AV, cT3 N1 M0	5 × 5, MRF–	LAR, no stoma; air leak reinforced by sutures	Yes	pT3 N1	W/M	0†	19 months LR (multifocal) + M (pulmonary). Also previous 10 months M (hepatic)	Work‐up to plan treatment for LR + M (pulmonary)	Alive at 22 months
12	M, 73, 10 cm AV, cT3 N1 M0	5 × 5, MRF–	LAR + diversion	No	pT3 N1	W/M	7	6 months LR (unifocal) + M (pulmonary, peritoneal, brain)	Palliative	Alive at 18·5 months

* After neoadjuvant treatment if applicable.

† Perineural growth. MRF, mesorectal fascia; CRM, circumferential resection margin; AV, anal verge; MRF–, MRF not threatened; LAR, low anterior resection; W/M, well to moderate; LR, local recurrence; M, distant metastasis; APR, abdominoperineal resection; IORT, intraoperative radiotherapy; CRT, chemoradiotherapy; TME, total mesorectal excision; 5 × 5, short‐course radiotherapy (RT) 5 × 5 Gy; CRS + HIPEC, cytoreductive surgery and hyperthermic intraperitoneal chemotherapy.

### Treatment of recurrences

Of the 12 patients with local recurrence, five with unresectable and/or systemic disease received palliative treatment. Six patients had local exenterative surgery with curative intent. Four patients underwent exenteration (1 combined with intraoperative radiotherapy (IORT)), one had abdominoperineal excision with IORT and one had cytoreductive surgery with hyperthermic intraperitoneal chemotherapy as salvage surgery. At the time of writing, the final patient was receiving further chemoradiotherapy before salvage surgery.

### Risk factors for recurrence

Risk factors for recurrence were identified by univariable logistic regression analysis. Prognostic factors associated with local recurrence (12 patients) were: positive CRM (odds ratio (OR) 11·67; *P* = 0·006), intraoperative complication (OR 7·00; *P* = 0·005), (y)pT3 category (OR 6·02; *P* = 0·025) and pelvic sepsis (OR 4·12; *P* = 0·029) (*Table* *S1*, supporting information). Risk factors associated with multifocal recurrence (8 patients) were: intraoperative complication (OR 12·11; *P =* 0·013), positive CRM (OR 9·00; *P* = 0·022), pathological N‐positive status (OR 6·88; *P* = 0·022), (y)pT3 category (OR 3·34; *P* = 0·150) and pelvic sepsis (OR 5·59; *P* = 0·023) (*Table* *S2*, supporting information).

### Proctoring effect

There were four patients with local recurrence among the first five proctored TaTME procedures per centre (4 of 60 overall) and eight occurred in the second five proctored TaTME procedures (8 of 60) (*P* = 0·362). Clinicopathological outcomes for the first and second five procedures per centre were an intraoperative complication rate of 3 *versus* 5 per cent respectively, an anastomotic leakage rate of 19 *versus* 16 per cent, and involved CRM rate of 2 *versus* 8 per cent.

### Comparative case‐matched analysis of transanal *versus* laparoscopic total mesorectal excision

To focus on the procedure itself rather than pathological risk factors for local recurrence, case‐matched pairing of patients with good‐quality specimens and no CRM involvement yielded two groups of 109 patients with similar baseline characteristics, abdominoperineal resection rate and incidence of anastomotic leakage (*Table* *S3*, supporting information). The pathological outcomes were comparable in terms of stage, and no patient in either matched group had a non‐radical resection or incomplete specimen. The overall local recurrence rate was higher for TaTME than laparoscopic TME: 8·3 per cent (nine patients) and 1·8 per cent (2) respectively.

### Long‐term outcomes of four hospitals with experience of more than 45 procedures

A prolonged cohort from four hospitals with experience of more than 45 procedures included a total of 266 patients who underwent TaTME for primary rectal cancer. Median follow‐up was 23·8 (range 1·0–62·4) months. The crude local recurrence rate was 15·0 per cent after the first ten procedures in each centre, 4·2 per cent after procedures 11–40, and 3·8 per cent for procedure 41 onwards (*Table* 6). Overall, 15 patients (5·6 per cent) in this cohort of 266 patients who underwent TaTME developed local recurrence.

**Table 6:** Local recurrence according to number of transanal total mesorectal excision procedures at each centre in prolonged cohort

	Local recurrence rate			
	Procedures 1–10	Procedures 11–40	Procedures ≥ 41	Total
Centre A	2 of 10	2 of 30	0 of 31	4 of 71 (6)
Centre B	1 of 10	2 of 30	3 of 28	6 of 68 (9)
Centre C	2 of 10	0 of 30	1 of 7	3 of 47 (6)
Centre D	1 of 10	1 of 30	0 of 40	2 of 80 (3)
Overall	6 of 40 (15)	5 of 120 (4·2)	4 of 106 (3·8)	15 of 266 (5·6)

Values in parentheses are percentages.

## Discussion

In this study, the local recurrence rate during the learning curve was 10·0 per cent, despite the low positive CRM rate and the presence of a structured training pathway, including on‐site proctoring. The multifocal pattern of recurrence seemed to be substantially different from that after abdominal TME (open, laparoscopic or robotic) and confirmed the pattern encountered in Norway^15^, which calls for further evaluation of the safety of TaTME. TaTME has been shown to be a difficult technique with a relatively long learning curve and associated morbidity^10^. Therefore, it was expected that some learning curve‐related problems would be encountered in the present cohort, despite the presence of a structured training pathway aimed at minimizing harm during implementation. The effect of the learning curve is demonstrated by the relatively high rate of anastomotic leakage and relatively high rate of local recurrences in the longer term. The present cohort size in each centre was inadequate for cumulative sum analysis with the endpoint local recurrence, but an increased recurrence rate among the first ten patients was clearly shown. This could reflect difficulties with poor execution of the technique causing unwanted tumour spillage. These data also demonstrate that the structured training as set out in this programme was not capable of diminishing all adverse outcomes, and should therefore be made more extensive for centres implementing this technique in the future. Proctoring of more than ten procedures should be advised until proficiency is met according to independent competency assessment using video analysis^22^.

Execution of the procedure rather than the technique itself may explain the observed recurrences. This is supported by the results of univariable analysis, which identified intraoperative events as the biggest risk factor. Two expert centres reported a 3‐year local recurrence rate of 2·0 per cent^9^. In the present study, long‐term outcomes from four centres with experience of more than 45 TaTME procedures after training indicated that the first ten procedures (early experience) are more at risk of local recurrence than the following 30. The 4·0 per cent local recurrence rate achieved after exclusion of the first ten procedures at each centre is more in line with the results reported by Hol and colleagues^9^ for the two expert centres starting this technique in the Netherlands. Longer follow‐up is needed to confirm the present recurrence rates, which should be interpreted with caution owing to inclusion of more challenging cases^23^.

The learning curve for implementation of new surgical techniques and its influence on long‐term oncological outcome is an important issue. Data are scarce, but a study of laparoscopic TME surgery demonstrated a significantly higher recurrence rate among the first 100 procedures compared with the following 200 (10·5 *versus* 4·9 per cent respectively)^24^. Robotic‐assisted TME surgery is being implemented worldwide, but data on the learning curve have focused on duration of operation, involved CRM rates and/or complications, and not on long‐term recurrence rates. A series by Polat and co‐workers^25^, reporting the first 77 procedures, documented a recurrence rate of 9·5 per cent despite a relatively low positive margin rate. This relatively high local recurrence rate was probably related to suboptimal technical execution within the learning curve.

The full report of the National Norwegian audit^16^ of 157 TaTME procedures revealed 12 local recurrences (7·6 per cent) after a median follow‐up of 19 months, with an estimated local recurrence rate of 11·6 per cent at 2·4 years according to Kaplan–Meier analysis. Wasmuth and colleagues^16^ stated that TaTME was responsible for the increased local recurrence rate, and that poor outcome could not be attributed to the learning curve effect because several of these recurrences occurred late in the series. However, four high‐volume centres performed 152 procedures over 4 years, which breaks down to an average annual volume of 9·5 procedures. This raises the question of whether the learning curve had been completed owing to the low exposure. A high rate of positive margins despite low tumour stage, the high rate of permanent stomas and perioperative morbidity may be indicative of suboptimal TaTME procedures. An unsupervised learning curve without proctoring, as shown by experienced single‐port surgeons, takes over 40 procedures^10^, ^11^.

The crucial difference in the TaTME technique is the endoluminal approach and potential direct contact with the tumour, whereas in the other abdominal techniques distal closure is assured by stapling below the tumour^26^. Poor tumour handling and inadequate closure of the lumen by failing purse strings could lead to tumour cells spilling into the pelvic dissection area during the procedure causing (multifocal) recurrences. This could be a similar mechanism to that described in early reports of laparoscopy demonstrating port‐site metastasis^27^. Careful evaluation led to the acknowledgement of tumour cell aerosolization combined with a chimney effect at the trocar sites. After implementation of sufficient training and clinical trials, it has now been proven that laparoscopy is safe when executed proficiently.

The multifocal local recurrence shown in this series and reported by Larsen and co‐workers^15^ seems to be a new pattern. In the Dutch TME trial^28^, the multifocality of recurrences was not evident on review of the imaging of patients with local recurrence. Other data regarding the incidence of multifocal local recurrences are scarce; large trials have not reported multifocality as a separate entity. In the present study, seven of 12 patients with local recurrence developed distant metastasis, similar to rates found in the Dutch TME^29^ and COLOR II^21^ trials, in which 50–60 per cent of patients with local recurrence also had distant metastasis. The question remains whether recurrence is related to the biology of the cancer rather than the surgical technique driving distant haematogenous spread of the disease^30^.

The explanation for both the high rate of multifocal recurrences and the local recurrence rate of 10·0 per cent, despite a relatively low CRM positivity rate of 5·0 per cent in this implementation cohort, could be multifactorial. Theoretically, unsuccessful execution of a TaTME procedure might result in inadequate purse‐string closure of the lumen. During the subsequent pelvic dissection, spilled tumour cells might be scattered as a result of the continuous high‐flow insufflation used in the dissection area in TaTME, leading to multifocal local recurrence. A high rate of positive bacterial cultures during TaTME, as reported by Velthuis and colleagues^31^, might provide support for this hypothesis. The authors have preliminary data showing that cancer cells can be cultured from rectal wash‐out (J. Tuynman; unpublished observation). Although the exact aetiology remains to be proven, all COLOR III sites have been instructed to secure the purse‐string closure with a second over‐running suture after the rectotomy with a secondary wash‐out^32^. Intraoperative perforation of the rectal tube in conventional TME might be regarded as a similar mechanism whereby tumour cells can seed in the pelvic cavity. In the present risk analysis, occurrence of intraoperative complications was the strongest predictor of multifocal local recurrence and second strongest for overall local recurrence. A previous study by Eriksen and colleagues^33^ showed a tremendous negative impact of perforation on 5‐year local recurrence, with the incidence rising from 9·9 per cent to 28·8 per cent in the presence of perforation (*P* < 0·001). The relatively high rate of pelvic sepsis (17·5 per cent) in the present learning curve cohort might also have contributed to the increased recurrence rate. A consistent hypothesis is that pelvic sepsis leads to an increased inflammatory reaction, and increased levels of growth factors associated with stimulation of adhesion and seeding of tumour cells^34^, ^35^, ^36^.

A potential weakness of this cohort study is the possible inclusion of some patients with advanced‐stage disease in the learning curve cohort. Overall, selection bias could be present within these data, but all patients who underwent TaTME for primary rectal cancer were included consecutively and the data were audited externally by an independent clinical researcher. Furthermore, case‐matched analysis of TaTME and laparoscopic TME procedures, excluding CRM‐positive and T4 tumours, demonstrated that TaTME during the learning curve was the only risk factor for local recurrence and not the pathology, showing that case selection was not an issue in the present cohort. Video analysis with surgical quality assessment could have revealed potential risk features for local recurrence. Quality assessment of every procedure is the central ingredient in the current COLOR III trial^22^, in which all data including MRI and the entire video of each procedure are captured centrally.

As stated in the IDEAL framework, a new innovation or technique should be evaluated stepwise, and not be implemented broadly before standardized indications and procedures have been developed. In this way, adverse effects and consistent outcomes can be established during the learning curve, which new centres can set as a benchmark^37^. The surgical community should focus on demonstrating oncological safety rather than surrogate endpoints for new innovative surgical techniques for patients with cancer. High‐quality data accrual in a clinical (randomized) trial is key, including establishing a safety commission and frequent external data monitoring^38^. The international TaTME guidance also states that TaTME should be implemented only in centres with a high volume of TME practice and with adequate training, including individual proctoring^2^.

## Contributors

Other proctors in the training pathway: H. B. A. C. Stockmann, R. C. L. M. Vuylsteke (Spaarne Hospital, Hoofddorp); P. G. Doornebosch (IJsselland Hospital, Cappelle aan den IJssel).

## Supporting information


**Table S1** Univariate analysis of risk factors for local recurrences
**Table S2** Univariate analysis of risk factors for Multifocal Local recurrence
**Table S3** Case matched analysis TaTME versus LapTME local recurrences
