# Supplementary material for: Locoregional recurrences after transanal total mesorectal excision of rectal cancer during implementation
Source: Br J Surg. 2020 Apr 4;107(9):1211–20. doi: 10.1002/bjs.11525 (PMC7496604; doi:10.1002/bjs.11525)
Supplement: Supplementary file 1 — Table S1 Univariate analysis of risk factors for local recurrences Table S2 Univariate analysis of risk factors for Multifocal Local recurrence Table S3 Case matched analysis TaTME versus LapTME local recurrences [file BJS-107-1211-s001.docx]

**BJS11525**

**Locoregional recurrences after transanal total mesorectal excision of rectal cancer during implementation**

S. E. van Oostendorp, H. J. Belgers, B. T. Bootsma, J. C. Hol, E. J. T. H. Belt, W. Bleeker, F. C. Den Boer, A. Demirkiran, M. S. Dunker, H. F. J. Fabry, E. J. R. Graaf, S. J. Oosterling, G. D. Slooter, D. J. A. Sonneveld, A. K. Talsma, H. L. Van Westreenen, M. Kusters, R. Hompes, H. J. Bonjer, C. Sietses and J. B. Tuynman

| **Table S1 Univariate analysis of risk factors for local recurrences** | | |  |  |  |
| --- | --- | --- | --- | --- | --- |
|  |  | Total events: 12 |  |  |  |
| Variabele |  | event/ total | OR | 95% CI | P |
| CRM involved | no | 9 / 114 | *ref* |  |  |
|  | yes | 3 / 6 | *11.667* | *2.050 - 66.408* | ***0.006*** |
| Intraoperative complication * | no | 10 / 115 | *ref* |  |  |
|  | yes | 2 / 5 | *7.000* | *1.044 - 46.949* | ***0.045*** |
| (y)pT-stage | 0-2 | 2 / 61 | *ref* |  |  |
|  | 3 | 10 / 59 | *6.020* | *1.259 - 28.786* | **0.025** |
| Pelvic sepsis | no | 7 / 99 | *ref* |  |  |
|  | yes | 5 / 21 | *4.107* | *1.160 - 14.544* | ***0.029*** |
| pN stage | negative | 5 / 80 | *ref* |  |  |
|  | positive | 7 / 40 | *3.182* | *0.941 - 10.761* | 0.063 |
| Chemoradiotherapy | no | 6 / 84 | *ref* |  |  |
|  | yes | 6 / 36 | *2.600* | *0.777 - 8.696* | *0.121* |
| Height | ≥6 cm from AV | 5 / 66 | *ref* |  |  |
|  | <6 cm from AV | 7 / 54 | *1.817* | *0.542 - 6.088* | 0.333 |
| Sex | female | 4 / 29 | *ref* |  |  |
|  | male | 8 / 91 | *0.602* | *0.167 - 2.168* | *0.438* |
| MRF+ (after RT) | no | 12 / 114 | *ref* |  |  |
|  | yes | 0 / 6 | **** |  |  |
| Specimen quality | complete | 12 / 107 | *ref* |  |  |
|  | nearly complete | 0 / 13 | **** |  |  |

OR = Odds ratio, MRF= mesorectal fascia, AV= anal verge, CRM= circumferential resection margin, RT = radiotherapy,

* Compostie of either intra operative perforation (n=0), purse string failure(n=1), reinforcement of anastomosis required (n=3)

| **Table S2 Univariate analysis of risk factors for Multifocal Local recurrence** | | |  |  |  |
| --- | --- | --- | --- | --- | --- |
|  |  | Total events: 8 |  |  |  |
| Variabele |  | event/ total | OR | 95% CI | P |
| Intraoperative complication * | no | 6 / 115 | *ref* |  |  |
|  | yes | 2 / 5 | *12.111* | *1.691 - 86.752* | **0.013** |
| CRM involved | no | 6 / 114 | *ref* |  |  |
|  | yes | 2 / 6 | *9.000* | *1.365 - 59.335* | **0.022** |
| pN stage | negative | 2 / 80 | *ref* |  |  |
|  | positive | 6 / 40 | *6.882* | *1.321 - 35.844* | **0.022** |
| Pelvic sepsis | no | 4 / 99 | *ref* |  |  |
|  | yes | 4 / 21 | *5.588* | *1.273 - 24.522* | **0.023** |
| (y)pT-stage | 0-2 | 2 / 61 | *ref* |  |  |
|  | 3 | 6 / 59 | *3.340* | *0.646 - 17.263* | **0.150** |
| Chemoradiotherapy | no | 4 / 84 | *ref* |  |  |
|  | yes | 4 / 36 | *2.500* | *0.589 - 10.607* | 0.214 |
| Height | ≥6 cm from AV | 3 / 66 | *ref* |  |  |
|  | <6 cm from AV | 5 / 54 | *2.143* | *0.488 - 9.406* | 0.313 |
| Sex | female | 2 / 29 | *ref* |  |  |
|  | male | 6 / 91 | *0.953* | *0.182 - 5.001* | 0.955 |
| MRF+ (after RT) | no | 8 / 114 | *ref* |  |  |
|  | yes | 0 / 6 | **** |  |  |
| Specimen quality | complete | 8 / 107 | *ref* |  |  |
|  | nearly complete | 0 / 13 | **** |  |  |

OR = Odds ratio, MRF= mesorectal fascia, AV= anal verge, CRM= circumferential resection margin, RT = radiotherapy,

* Compostie of either intra operative perforation (n=0), purse string failure(n=1), reinforcement of anastomosis required (n=3)

| **Table S3 Case matched analysis TaTME versus LapTME local recurrences** | | | | | | |
| --- | --- | --- | --- | --- | --- | --- |
|  | *Unmatched* | | | *Matched* | | |
|  | **TaTME** | **lapTME** |  | **TaTME** | **lapTME** |  |
|  | n= 120 | n= 699 | *p-value* | Proctor (n=109) | COLOR II (n=109) | *p-value* |
| Sex (Male) | 91 (75.8) | 448 (64.1) | 0.012 | 82 (75.2) | 82 (75.2) | 1,000 |
| Age (Mean ±SD) | 65.4 (±9.6) | 66.9 (10.4) | 0.164 | 65.2 (9.7) | 66.2 (11.3) | 0.355 |
| Height cm from AV (mean SD) | 6.9 (3.1) | 7.8 (4.0) | 0.000 | 7.1 (2.9) | 7.4 (3.0) | 0.412 |
| No neoadjuvant treatment | 43 (35.8) | 222 (31.8) | 0.897 | 40 (36.7) | 40 (36.7) | 1,000 |
| Chemoradiotherapy | 36 (30.0) | 192 (31.5) | 0.742 | 33 (30.3) | 33 (30.3) | 1,000 |
| Short course RT | 41 (34.2) | 192 (27.5) | 0.133 | 36 (33.0) | 30 (27.5) | 0.376 |
|  |  |  |  |  |  |  |
| LAR TaTME | 110 (91.7) | 490 (70.1) | 0.000 | 101 (92.7) | 101 (92.7) | 1,000 |
| ISR-APR TaTME | 10 (8.3) | 200 (28.6) |  | 8 (7.3) | 8 (7.3) | 1,000 |
| Anastomotic Leakage | 17 (17.3) $ | 48 (6.9) | 0.006 | 13 (12.9) | 13 (13.1) | 0.956 |
|  |  |  |  |  |  |  |
| *(y)pTx* | 0 (0.0 | 10 (1.5)* | 0.001 | 0 (0.0) | 0 (0.0) | 0.253 |
| *(y)pT0* | 11 (9.2) | 19 (2.8)* |  | 10 (9.2) | 3 (2.8) |  |
| *(y)pT1* | 16 (13.3) | 52 (7.7)* |  | 13 (11.9) | 13 (11.9) |  |
| *(y)pT2* | 34 (28.3) | 232 (34.2)* |  | 34 (31.2) | 38 (34.9) |  |
| *(y)pT3* | 59 (49.2) | 343 (50.6)* |  | 52 (47.7) | 55 (50.5) |  |
| *(y)pT4* | 0 (0.0) | 22 (3.2)* |  | 0 (0.0) | 0 (0.0) |  |
| CRM+ | 6 (5.0) | 21 (3.6)** | 0.425 | 0 (0.0) | 0 (0.0) | 1,000 |
| DRM+ | 0 (0.0) | 13 (2.0)*** | 0.240 | 0 (0.0) | 0 (0.0) | 1,000 |
| R1 | 6 (5.0) | 31 (5.2)**** | 0.957 | 0 (0.0) | 0 (0.0) | 1,000 |
| Specimen by Quirke |  |  |  |  |  |  |
| complete | 107 (89.2) | 589 (88.4)***** | 0.140 | 97 (89.0) | 98 (89.9) | 0.296 |
| nearly complete | 13 (10.8) | 58 (8.7) |  | 12 (11.0) | 9 (8.3) |  |
| incomplete | 0 (0.0) | 19 (2.9) |  | 0 (0.0) | 2 (1.8) |  |
|  |  |  |  |  |  |  |
| Local recurrence overall | 12 (10.0) | 31 (5.0) | 0.001 | 9 (8.3) | 2 (1.8) | 0.004 |

$ only patients with anastomosis (n=98)

*denominator=678 (21 missing data)

**denominator=588 (699-111 missing data)

***denominator=627 (699-72 missing data)

****denominator=600 (699-99 missing data)

*****denominator=666 (699-33 missing data)

**Abbreviations:** M= Male, F= Female, MRF+= mesorectal fascia threatened, CRT= chemoratiotherapy, RT= Short course radiothearpy 5x5 Gy,LAR= low anterior resection, ISR= Intersfincteric resection, APR= abdominoperineal resection, CRM= circumferential resection margin, DRM= distal resection margin, R1 = Irradical resection, LR - local recurrence, Mo= month, FU = follow-up
